# Differentially Infiltrated Identification of Novel Diagnostic Biomarkers Associated with Immune Infiltration in Nasopharyngeal Carcinoma

**DOI:** 10.1155/2022/3934704

**Published:** 2022-11-17

**Authors:** Pei Gao, Wuhao Lu, Shousen Hu, Kun Zhao

**Affiliations:** Department of Otolaryngology Head and Neck Surgery, The First Affiliated Hospital of Zhengzhou University, No. 1 Jianshe Road, Zhengzhou, 450052 Henan, China

## Abstract

**Background:**

The prognostic value of tumor-infiltrating immune cells has been widely studied in nasopharyngeal carcinoma (NPC). However, the role of tumor-infiltrating immune cells in the diagnosis of NPC has not been fully elucidated. Thus, tumor-infiltrating immune cell-related biomarkers in the diagnosis of NPC patients were explored in the current study.

**Methods:**

Gene expression profiles of NPC patients were downloaded from the Gene Expression Omnibus (GEO) database. Differentially infiltrating immune cells (DDICs) between NPC and control samples were analyzed by the CIBERSORT algorithm. Weighted gene coexpression network analysis (WGCNA) was performed to screen hub genes significantly correlated with DDIC. Gene Ontology (GO) and Kyoto Encyclopedia of Genes and Genomes (KEGG) pathway enrichment analyses of hub genes were performed with R package clusterProfiler. The diagnostic value of hub genes was evaluated by receiver operating characteristic (ROC) curves. RT-qPCR was conducted to validate the expression patterns of diagnostic markers in NPC and adjacent control tissues. The correlations between diagnostic markers and immunomodulators were analyzed using the TISIDB. The protein-protein interaction (PPI) network based on immunomodulators significantly associated with diagnostic biomarkers was constructed and visualized by STRING. The functional enrichment analysis of genes in the PPI network was analyzed by the WebGestalt online tool.

**Results:**

The abundances of memory B cells, plasma cells, follicular helper T cells, activated NK cells, M0 macrophages, M1 macrophages, M2 macrophages, resting mast cells, and activated mast cells were significantly different between NPC and control samples. Dark orange was identified as the hub module, with a total of 371 genes associated with memory B cells, plasma cells, and M0 and M1 macrophages defined as hub genes, which were enriched into immune-related biological processes and pathways. *FCER2*, *KHDRBS2*, and *IGSF9* were considered diagnostic biomarkers with areas under ROC curves as 0.985, 0.978, and 0.975, respectively. Moreover, real-time reverse transcriptase-polymerase chain reaction (RT-qPCR) suggested that the expression patterns of *FCER2*, *KHDRBS2*, and *IGSF9* were consistent with the results in GEO datasets. TISIDB analysis revealed that *FCER2*, *KHDRBS2*, and *IGSF9* had a strong association with 8 immunoinhibitors (*BTLA*, *CD160*, *CD96*, *LAG3*, *PDCD1*, *TIGIT*, *CD244*, and *TGFB1*) and 11 immunostimulators (*CD27*, *CD28*, *CD40LG*, *CD48*, *ICOS*, *KLRC1*, *KLRK1*, *TMIGD2*, *TNFRSF13C*, *CXCR4*, and *C10* or *f54*). The PPI network implied that these 19 immunomodulators had interactions with other 50 genes. WebGestalt analysis demonstrated that 69 genes in the PPI network were enriched into cytokine-cytokine receptor interaction, NF-kappa B signaling pathway, and pathways in cancer.

**Conclusion:**

Our study identified novel diagnostic biomarkers and revealed potential immune-related mechanisms in NPC. These findings enlighten the investigation of the molecular mechanisms of tumor-infiltrating immune cells regulating NPC.

## 1. Introduction

Nasopharyngeal carcinoma (NPC) is a malignant tumor caused by epithelial cells of the nasopharynx. It is one of the most common head and neck tumors, the global geographical distribution of which is extremely uneven that more than 70% of new cases occur in East and Southeast Asia [1]. Common clinical manifestations of NPC contain nasal congestion, epistaxis, ear blockage, hearing loss, diplopia, and headache. Radiotherapy is only the main treatment for early diseases, while chemotherapy combined with radiotherapy is an essential progress in the treatment of locally advanced diseases [2]. However, the mortality rate of NPC remains high, making the detection of preclinical specific markers for nasopharyngeal carcinoma particularly important for the early diagnosis and treatment of NPC [3]. There are still significant differences in outcomes among patients receiving similar treatments at the same stage, though the tumor-lymph node metastasis (TNM) cancer staging system provides useful criteria for recommending treatment strategies or assessing patient outcomes. Therefore, it is of great significance to explore novel diagnostic biomarkers for NPC patients to assess the NPC status or guide treatment.

The malignant phenotype of cancer is also defined by immune cells activated in the tumor microenvironment (TME) [4]. TME consists of immune cells, endothelial cells, mesenchymal cells, inflammatory mediators, and extracellular matrix molecules [5]. In TME, immune cells are a major type of nontumor component and can be applied to tumor diagnosis and prognosis assessment [6–9]. Besides, the tumor-infiltrating immune cells (TIICs) have a significant prognostic value in NPC, such as T cells, macrophages, dendritic cells, and mast cells [10, 11].

However, the diagnostic value and molecular mechanism of immune infiltration-related genes in NPC remain to be elucidated. Therefore, this study is aimed at exploring the possibility of TIIC-related genes as diagnostic markers for NPC, analyzing the pathways of their involvement in NPC immune regulation, and thus laying a theoretical foundation for the value of TIICs in NPC diagnosis.

## 2. Materials and Methods

### 2.1. Ethical Statement

From June 1, 2021, to November 1, 2021, 10 patients with NPC were selected from the throat and head and neck surgery at the First Affiliated Hospital of Zhengzhou University. All patients were confirmed as nasopharyngeal carcinoma by histology at the primary tumor site. All cases were nonkeratinizing squamous cell carcinoma. The patient did not receive radiotherapy or chemotherapy before the operation. Patients with World Health Organization (WHO) type II NPC were excluded. Fresh living cancer tissues and adjacent tissues of 10 nasopharyngeal carcinoma patients were collected during the operation. The basic clinical information of all patients is collected in Table 1. The study was approved by the central institutional review committee and ethics committee of the First Affiliated Hospital of Zhengzhou University (ethical approval number: 2021-KY-1024-002). This study was conducted following the provisions of the Declaration of Helsinki.

### 2.2. Data Source

Gene expression profiles were downloaded from GSE53819 and GSE12452 datasets. GSE53819 contained 18 NPC and 18 control noncancerous nasopharyngeal tissue (NCNT) samples and was used to identify diagnostic biomarkers. GSE12452 included 31 NPC and 10 NCNT samples and was applied to external validation of the expressions of diagnostic biomarkers between NPC and NCNT samples.

### 2.3. Estimation of Immune Cell Infiltration in NPC and NCNT Samples

CIBERSORT was performed to estimate the infiltrations of 22 immune cells in NPC and NCNT samples, involving naive B cells, memory B cells, plasma cells, CD8 T cells, naive CD4 T cells, resting memory CD4 T cells, activated memory CD4 T cells, follicular helper T cells, regulatory T cells (Tregs), gamma delta T cells, resting NK cells, activated NK cells, monocytes, M0 macrophages, M1 macrophages, M2 macrophages, resting dendritic cells, activated dendritic cells, resting mast cells, activated mast cells, eosinophils, and neutrophils [12].

### 2.4. WGCNA Analysis

A sample clustering tree map was first constructed to detect and eliminate outliers. Then, WGCNA was performed based on the gene expression profiles from the GSE53819 dataset and sample traits (differentially infiltrating immune cells between NPC and NCNT samples) [13]. Besides, *β* from 1 to 30 was calculated using the pick soft threshold function of WGCNA to select the best soft threshold. Based on the selected soft threshold, the adjacency matrix was converted to a topological overlap matrix to construct the network, and the gene dendrogram and module color were established with the degree of dissimilarity. Additionally, the initial module was further divided by dynamic tree cutting, and similar modules were merged. The Pearson correlation coefficient between the module eigengenes and sample traits was calculated to reveal the most relevant module (hub module) associated with sample traits [14].

### 2.5. Identification of Hub Genes Associated with Immune Infiltration

The genes in the hub module were further screened using the module membership (MM) > 0.8 and |*gene* *significance* (*GS*)| > 0.5. Hub genes were identified by overlapping genes associated with differentially infiltrating immune cells using Venn software online (http://bioinformatics.psb.ugent.be/webtools/Venn/) [15]. Next, functional enrichment analyses including GO pathway analysis and KEGG pathway analysis were performed with R package clusterProfiler [16]. The top five GO terms and the top three KEGG pathways were visualized in a circle plot through the R package GO plot, and *p* adjust value > 0.5 was considered significant difference.

### 2.6. Identification of Diagnostic Markers in NPC

R package limma [17] was applied to identify differentially expressed hub genes between NPC and NCNT samples with |log2 (fold change)| > 2 and FDR < 0.05. DEGs were visualized in the volcano plot. The diagnostic value of differentially expressed hub genes was evaluated using ROC curves. Hub genes with the areas under the ROC curves > 0.7 were identified as diagnostic biomarkers with high accuracy in NPC. The correlations between diagnostic markers and immunomodulators (immunoinhibitors and immunostimulators) were calculated using the TISIDB (http://cis.hku.hk/TISIDB/) to better understand the relationship between diagnostic markers and immune response [18]. A PPI network based on diagnostic biomarker-related immunomodulators was constructed using the STRING database [19]. Moreover, the functional enrichment analysis of genes in the network was analyzed by the WebGestalt online tool [20].

### 2.7. The Real-Time Reverse Transcriptase-Polymerase Chain Reaction (RT-qPCR)

Total RNA of NPC (*N* = 10) and adjacent control tissues (*N* = 10) were extracted by Nuclezol LS RNA Isolation Reagent (ABP Biosciences Inc., China). After the concentration and purity of RNA were detected, qualified RNA was used for reverse transcription with SureScript-First-strand-cDNA-synthesis-kit (GeneCopoeia, USA). Then, qPCR on a CFX96 real-time PCR system (Bio-Rad, USA) was performed using BlazeTaq™ SYBR® Green qPCR Mix 2.0 (GeneCopoeia, USA) under the thermal cycling conditions: 40 cycles at 95°C for 30 s, 95°C for 10 s, 60°C for 20 s, and 72°C for 30 s. Besides, gene expressions were calculated with the 2^-△△Ct^ method [21]. The primer sequences used in the current study are listed in Table 2.

### 2.8. Statistical Analysis

All data were analyzed by R (version 4.0.0). Comparisons between the two groups were calculated using the Wilcoxon test. *p* value < 0.05 indicated statistical significance unless specified.

## 3. Results

### 3.1. Nine Immune Cells Were Differential Infiltration between NPC and NCNT

**Figure 1:**
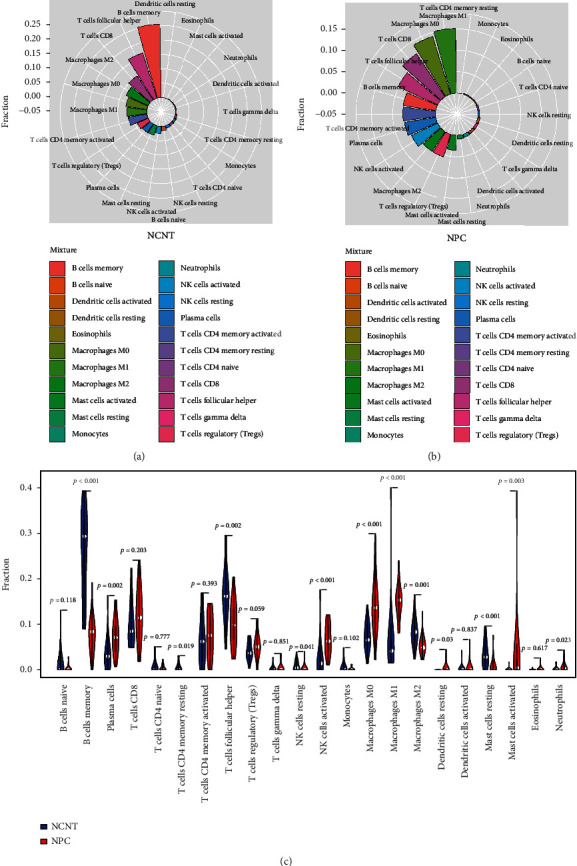
(a) The distribution of the immune cells in NCNT. (b) The distribution of the immune cells in NPC. (c) Comparison of 9 immune cells with significant difference between NPC and NCNT (*p* < 0.01).

The composition of infiltrating immune cells in NPC and NCNT was first detected and compared. It was revealed that the top two most abundant immune cells were memory B cells and follicular helper T cells in NCNT (Figure 1(a)), while the proportions of M0 and M1 macrophage infiltration were the highest in NPC (Figure 1(b)). The comparison suggested that the infiltration levels of memory B cells, resting memory CD4 T cells, follicular helper T cells, resting NK cells, M2 macrophages, and resting mast cells were significantly higher in NCNT, while the abundances of plasma cells, activated NK cells, M0 macrophages, M1 macrophages, resting dendritic cells, activated mast cells, and neutrophil infiltration in NPC were significantly boosted (Figure 1(c)). Memory B cells, follicular helper T cells, M0 macrophages, M1 macrophages, M2 macrophages, resting mast cells, activated mast cells, plasma cells, and activated NK cells with extremely significant differences between NPC and NCNT (*p* < 0.01, Figure 1(c)) were selected for the following WGCNA analysis.

### 3.2. WGCNA Identifies the Dark Orange Module as the Hub Immune Cell-Related Module

**Figure 2:**
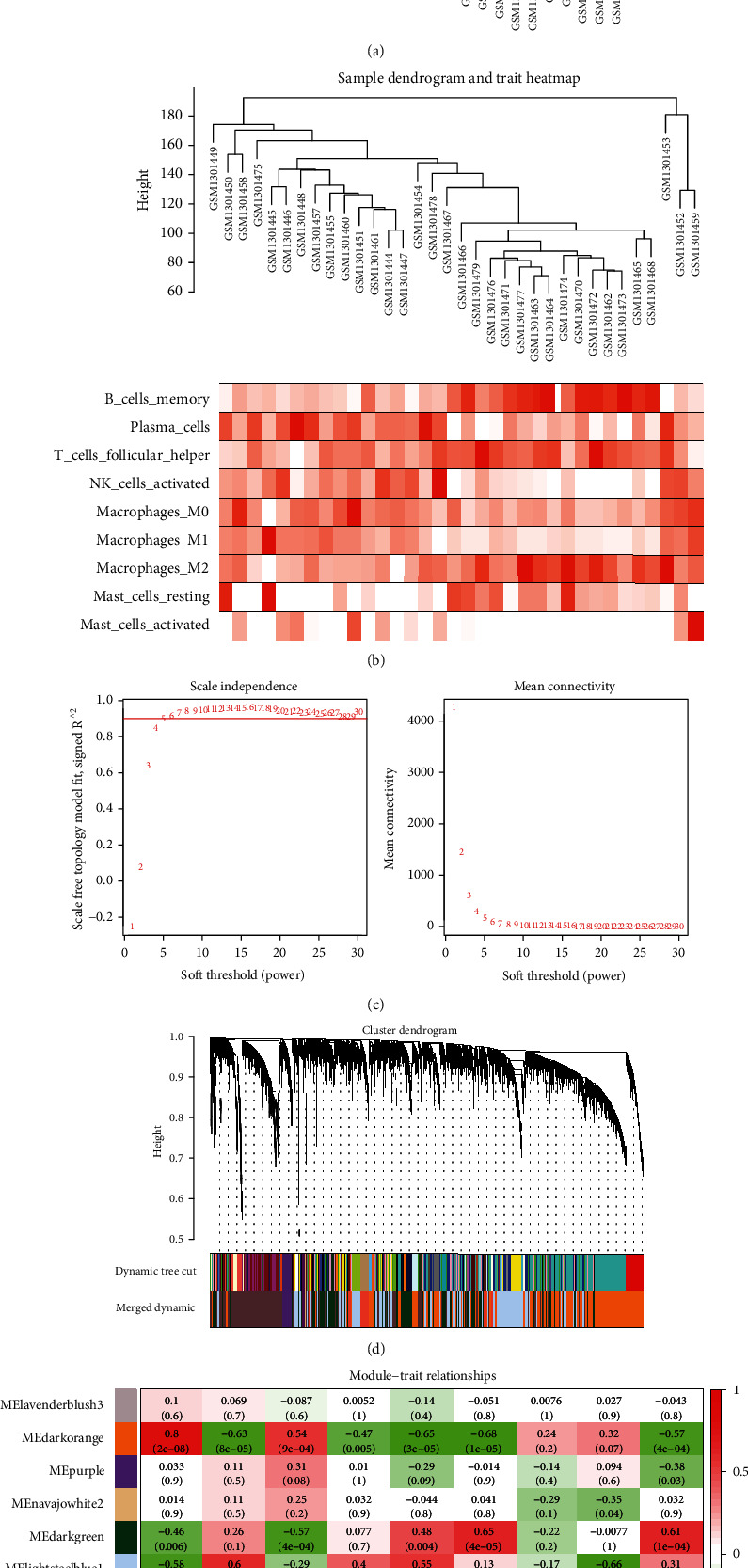
(a) The sample clustering result. (b) The sample dendrogram and trait heatmap. (c) The pick soft threshold function of WGCNA. (d) Cluster dendrogram. (e) Screening of the hub module associated with immune infiltration in NPC. Each row represents a color-coded module eigengene; each column represents a type of infiltrating immune cells. The number in each cell means the correlation coefficient and *p* value.

WGCNA was performed to screen the most relevant module associated with infiltrating immune cells. According to the sample clustering result, two outlier samples were detected and eliminated (Figure 2(a)), and then, the sample dendrogram and trait heatmap were established (Figure 2(b)). It was indicated by the pick soft threshold function of WGCNA that the optimal soft threshold power was 5, in which *R*^2^ was about 0.9 (Figure 2(c)). After similar modules were merged, ten modules from the coexpression network were identified (Figure 2(d)). As suggested by the module-trait relationships in Figure 2(e), the dark orange module was the most relevant module associated with memory B cells (Cor = 0.8, *p* < 0.01), plasma cells (Cor = −0.63, *p* < 0.01), M0 macrophages (Cor = −0.65, *p* < 0.01), and M1 macrophages (Cor = −0.68, *p* < 0.01) and was identified as hub module related to infiltrating immune cells.

### 3.3. Identification and Functional Enrichment Analysis of 371 Immune Cell-Related Hub Genes

**Figure 3:**
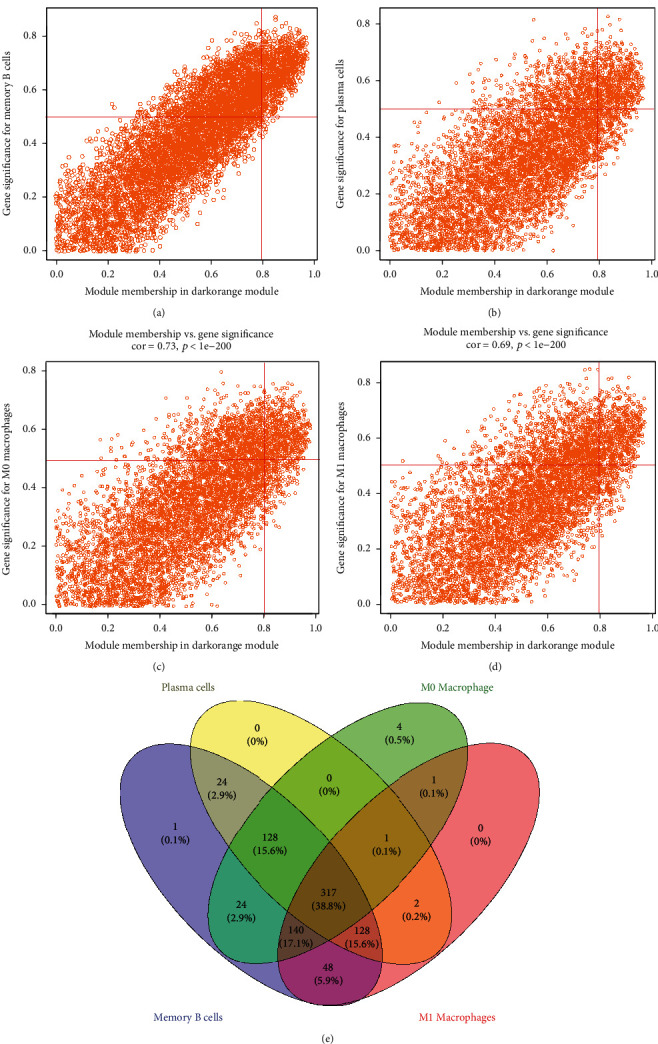
(a–d) Genes related to memory B cells, plasma cells, M0 macrophages, and M1 macrophages. (e) 371 hub genes associated with immune cell infiltration were identified.

**Figure 4:**
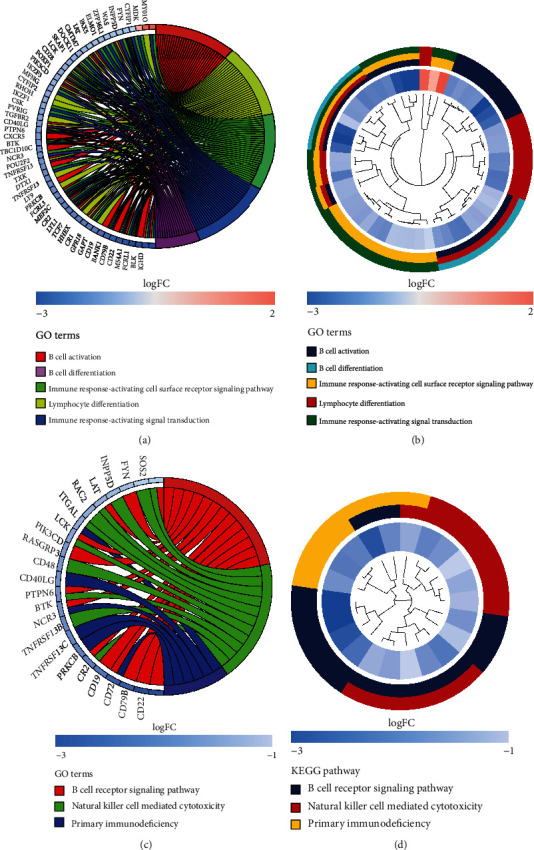
(a, b) B cell activation, lymphocyte differentiation, immune response activating cell surface receptor signaling pathway, immune response activating signal transduction, and B cell differentiation, and corresponding genes are visualized in these terms. (c, d) The first three pathways of KEGG, B cell receptor signaling pathway, natural killer cell-mediated cytotoxicity, primary immunodeficiency, and hub genes involved in these three pathways are shown in the circular diagram.

Thereafter, MM > 0.8 and |GS| > 0.5 were used to further screen hub genes in the dark orange module. A total of 810, 600, 615, and 637 genes were discovered to be correlated with memory B cells, plasma cells, and M0 and M1 macrophages, respectively (Figures 3(a)–3(d)). By overlapping these genes, 371 genes were obtained and identified as hub genes associated with immune cell infiltration (Figure 3(e)). GO and KEGG pathway analyses were conducted to investigate the biological function of hub genes. A total of 114 biological processes (BP), 6 cellular components (CC), 14 molecular functions (MF), and 18 KEGG pathways were significantly enriched (Table S1 and S2). As illustrated in Figures 4(a) and 4(b), the top five GO terms were B cell activation, lymphocyte differentiation, immune response-activating cell surface receptor signaling pathway, immune response-activating signal transduction, and B cell differentiation; the corresponding genes involved in these GO terms were visualized in the circle plot. The top three KEGG pathways were B cell receptor signaling pathway, natural killer cell-mediated cytotoxicity, primary immunodeficiency, and hub genes involved in the three pathways, as displayed in the circle plot (Figures 4(c) and 4(d)).

### 3.4. Identification of Diagnostic Biomarkers Associated with Immunomodulators

**Figure 5:**
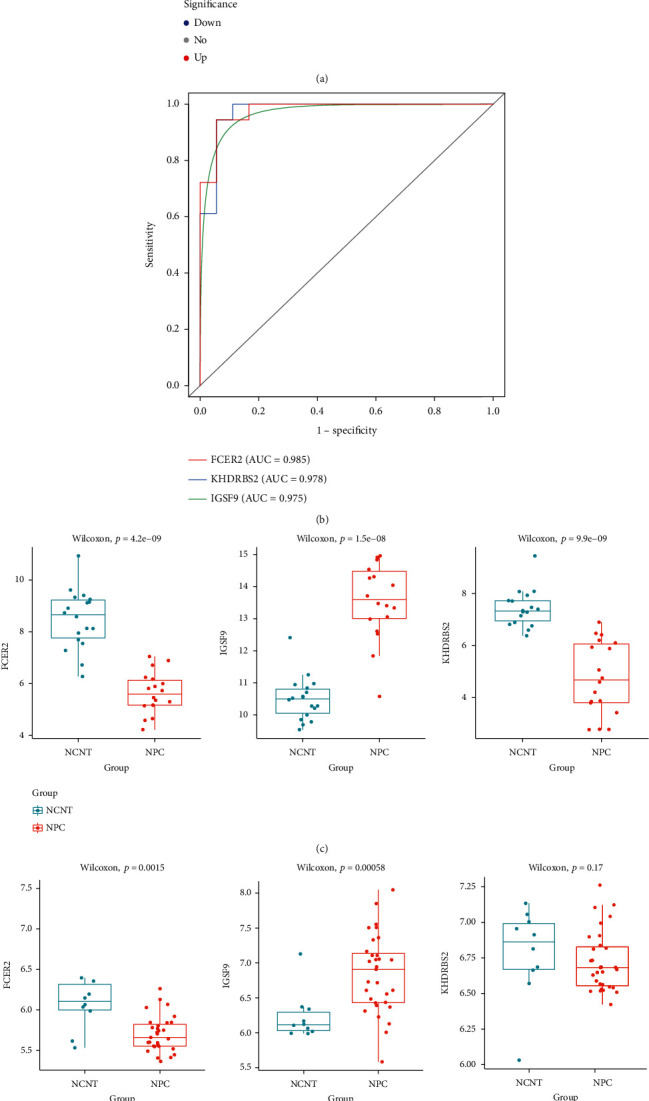
(a) Compared with NCNT, 7 hub genes were upregulated and 43 hub genes were downregulated in NPC samples. (b) The diagnostic effectiveness of candidate biomarkers was evaluated by the ROC curve. (c) Differential expression of candidate diagnostic markers in NPC and NCNT in the GSE53819 dataset. (d) In the GSE12452 dataset, the expression of candidate diagnostic biomarkers. (e) The expression levels of candidate genes isolated from NPC and NCNT patients were analyzed by RT-qPCR.

Next, the expressions of 371 hub genes between NPC and NCNT cells were compared to identify hub genes associated with NPC. A total of 50 differentially expressed hub genes were identified, including 7 upregulated and 43 downregulated hub genes in NPC samples related to NCNT ones (Figure 5(a)). ROC curves identified *FCER2*, *KHDRBS2*, and *IGSF9* with high diagnostic accuracy that the areas under the ROC curves for *FCER2*, *KHDRBS2*, and *IGSF9* were 0.985, 0.978, and 0.975, respectively (Figure 5(b)). Thus, *FCER2*, *KHDRBS2*, and *IGSF9* were identified as diagnostic biomarkers in NPC. The expression of *IGSF9* was significantly higher, while the expressions of *FCER2* and *KHDRBS2* were significantly lower in NPC samples compared with NCNT ones (Figure 5(c)). Consistent expression results were obtained in the external validation dataset GSE12452 (Figure 5(d)). Moreover, the expressions of *FCER2*, *KHDRBS2*, and *IGSF9* in vivo were detected by RT-qPCR. Similarly, the expression of *IGSF9* was significantly higher, while the expressions of *FCER2* and *KHDRBS2* were significantly lower in NPC samples compared with adjacent control ones (Figure 5(e)).

**Figure 6:**
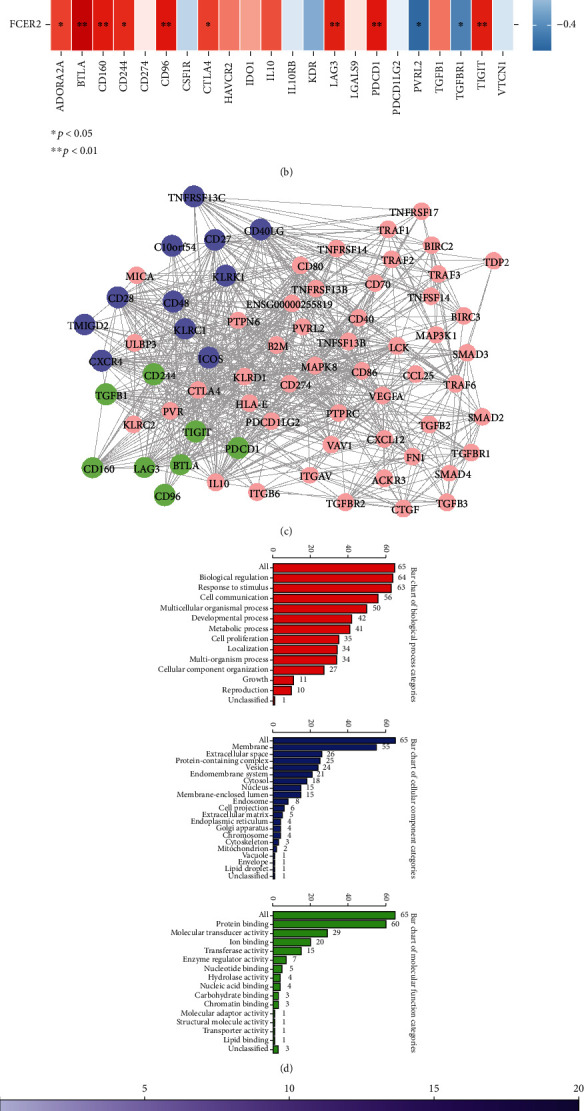
(a) The heatmap of correlations between immunostimulators and target genes. (b) The heatmap of correlations between immunoinhibitors and target genes. ^∗^ stands for *p* < 0.05, and ^∗∗^ stands for *p* < 0.01. (c) PPI network composed of 19 immunomodulators and 50 genes interacting with them. Purple represents 11 immune enhancers, green represents 8 immunosuppressants, and pink represents 50 mRNA. (d) Bar charts of biological process categories, cellular component categories, and molecular function categories. (e)Bubble plot of 69 genes significantly enriched cancer and immune-related biological processes and pathways.

Afterward, the relationship between diagnostic biomarkers and immunomodulators was investigated. Regarding immunostimulators in Figure 6(a), *FCER2* was strongly positively correlated with *CD27*, *CD28*, *CD40LG*, *CD48*, *ICOS*, *KLRC1*, *KLRK1*, *TMIGD2*, and *TNFRSF13C* (*r* > 0.6 and *p* < 0.05). *KHDRBS2* had strong positive correlations with *CD28*, *CD40LG*, *CD48*, *CXCR4*, and *KLRK1* (*r* > 0.6 and *p* < 0.05). *IGSF9* was strongly negatively associated with C10orf54 (*r* < −0.6 and *p* < 0.05). Concerning immunoinhibitors in Figure 6(b), *FCER2* was strongly positively correlated with *BTLA*, *CD160*, *CD96*, *PDCD1*, and *TIGIT* (*r* > 0.6 and *p* < 0.05). *KHDRBS2* had strong positive correlations with *BTLA*, *CD96*, and *PDCD1* (*r* > 0.6 and *p* < 0.05). *IGSF9* was strongly negatively associated with *CD244*, *PDCD1*, and *TGFB1* (*r* < −0.6 and *p* < 0.05). Moreover, a PPI network composed of above 19 immunomodulators and 50 genes interacting with them was constructed to further explore the interactions among immunomodulators (Figure 6(c)). By the WebGestalt online tool, these 69 genes were significantly enriched into cancer and immune-related biological processes and pathways, such as cell proliferation, cell communication, cytokine-cytokine receptor interaction, cell adhesion molecules, NF-kappa B pathway, natural killer cell-mediated cytotoxicity, T cell receptor signaling pathway, and pathways in cancer (Figures 6(d) and 6(e)).

## 4. Discussion

The growth of NPC tumor cells is regulated by surrounding tumor cells, various immune cells, fibroblasts, and endothelial cells [22]. It is of great clinical significance for early diagnosis, treatment, and prognosis to study the distribution and affinity of immune cells in the tumor microenvironment. Therefore, in this study, the landscape of infiltrating immune cells in NPC was detected, and *FCER2*, *KHDRBS2*, and *IGSF9* associated with immune were identified as diagnostic biomarkers of NPC.

In this study, memory B cells, follicular helper T cells, M0 macrophages, M1 macrophages, M2 macrophages, resting mast cells, activated mast cells, plasma cells, and activated NK cells were revealed to be differentially infiltrating between NPC and NCNT samples. Tumor-infiltrating B cells (B cells and memory B cells) participate in the genesis and development of NPC [22]. Gong et al. applied single-cell RNA sequencing of 66,627 cells from 14 patients with NPC and integrated clonotype identification on T and B cells. The findings suggested that the severe infiltration of dysfunctional and immunosuppressive T cells remarkably affected T cell immunity against NPC [23]. Liu et al. evaluated the potential prognostic value of tumor-infiltrating macrophages (TIMs) in patients with NPC [24]. TIMs can be divided into M1 and M2 subtypes following their phenotypes and functions. *CD68* is expressed by all TIMs, whereas *CD163* is a marker of the M2-like subpopulation. Additionally, *CD163+ TIMs* are predominantly correlated with NPC's poor prognosis, while total *CD68*+ *TIMs* are not associated with survival [25].

Deng et al. believed that tumor-related macrophages (TAMs) are a new target for the combined treatment of NPC to improve the efficiency of ICBs [26]. Our study verified that follicular helper T cells are one of the most abundant immune cells in NCNT. The proportion of infiltrating M0 and M1 macrophages was the highest in NPC. High expression of T cells in NCNT can be ascribed to low expression in NPC, which plays an inhibitory role in the development of NPC. The high degree of infiltration of giant cells in nasopharyngeal carcinoma indicates that the high expression of giant cells may be related to the prognosis of NPC, consistent with the previous literature that *CD163*+ giant and fine cells are associated with the poor prognosis of NPC. Mast cells are considered regulators of the tumor microenvironment. They can directly regulate the proliferation and invasion of tumor cells and indirectly help tumors by promoting tumor angiogenesis, remodeling the tumor microenvironment, and regulating immune response [27].

In addition to mast cells, NK cells are cytotoxic natural immune cells, which are specially employed to defend against tumor and virus-infected cells. NK cells can quickly recognize and dissolve mutant cells without antibody participation and early sensitization through the perforin-/granzyme-mediated cytotoxicity pathway or *Fas/FasL*-mediated apoptosis pathway [28]. Furthermore, the complex interaction between NK cells and the tumor microenvironment of NPC contributes to the prognosis of NPC [29]. Liao et al. discovered that the presence of CTL, Treg cells, neutrophils, and mast cells was associated with a poor prognosis of NPC, while a considerable number of tumor-infiltrating NK cells were correlated with a good prognosis of NPC. Meanwhile, the number of NK cells combined with mast cells can be used as a biomarker to predict the recurrence or distant metastasis of NPC [20]. Although Asians have a high content of resting mast cells and a good prognosis in lung adenocarcinoma [30], there are few studies on nasopharyngeal carcinoma. In this study, NK cells were activated, M0 macrophages, M1 macrophages, dendritic cells rested, and activated mast cells and neutrophils infiltrated in NPC. It was suggested that these cells are related to the occurrence and development of NPC, consistent with the view of Lu et al. [27].

The WGCNA analysis was conducted to find the genes related to these immune cells. A total of 371 key genes were revealed to be significantly associated with memory B cells, plasma cells, M0 macrophages, and M1 macrophages. Functional enrichment of those key genes demonstrated that they were involved in the B cell receptor signaling pathway. *CD40* and B cell receptors (BCRs) are simulated by *LMP1* and *LMP2*, respectively [31], and are tumor necrosis factor (TNF) receptors and key costimulatory receptors for B cells [32]. Kim et al. [33] reported that *LMP1* is required for EBV-mediated B lymphocyte transformation in EB Epstein-Barr virus-related NPC. *LMP1* can activate *PI3K/AKT* and *HIF-1α* signaling pathways in EBV-positive NPC cells and function in chemokine ligand 5- (CCL5-) mediated tumor angiogenesis [34]. In our study, it was hypothesized that the B cell receptor signaling pathway may be involved in the development and metastasis of EB virus-related NPC. Next, we will explore a lot of evidence about how these genes participate in cell signal transduction in NPC.

The ROC and RT-qPCR analyses revealed that *FCER2*, *KHDRBS2*, and *IGSF9* may play as potential biomarkers for NPC. There is little research on the pathogenesis of *FCER2* in NPC. In B cells, *FCER2* is a polypeptide gene encoded by *CD23 FCER2* microsatellite [35]. The single nucleotide polymorphism of *FCER2* (19p13 locus) indicates that the genetic changes of this gene may also impact the level of IgE [36, 37]. IgE receptor IIFC fragment (*FCER2*) is expressed in macrophages, eosinophils, B cells, and platelets. *FCER2* is involved in the regulation of IgE response, the growth and differentiation of T and B cells, cell adhesion, and antigen presentation [38–40]. As mentioned previously, memory B cells and M2 macrophages infiltrate better in NCNT than in NPC. Concurrently, the expression of *FCER2* in NPC samples was significantly lower than that in adjacent tissues, reflecting that *FCER2* is a downregulated gene of NPC. This would correspond to the degree of infiltration of memory B cells and M2 macrophages.

*KHDRBS2* encodes an RNA binding protein involved in regulating alternative splicing. It can function as an adaptor protein during mitosis and interact with the product of the EBV early gene *BSLF2/BMLF1* [41]. A comprehensive study measured EBV copy numbers in an array of lymphoblastoid cell lines derived from more than 1700 individuals and identified multiple genetic variants pointing to putatively relevant genes related to EBV infection, such as *KHDRBS2* [42]. Our study demonstrated that the expression of *KHDRBS2* significantly decreased in NPC samples than that in NCNT samples. It was previously reported that *KHDRBS2* is associated with the coding gene of the EBV. Thus, *KHDRBS2* may be involved in cell cycle control and transcription involving cell signaling pathways associated with the occurrence of EBV-related NPC. Moreover, the low expression of *KHDRBS2* promotes the occurrence and development of NPC.

*IGSF9* belongs to the immunoglobulin superfamily and plays a key role in inhibiting synaptic development by regulating calmodulin-like activity. Huang et al. confirmed that *IGSF9* promoted the proliferation, migration, and invasion of NPC cells in vitro. *IGSF9* may be a prognostic gene promoting the invasion and metastasis of NPC cells. The expression of *IGSF9* in NPC cells may be affected by hypoxia [43]. Our results suggested that *IGSF9* was highly expressed in NPC tissues compared with NCNT. Meanwhile, *IGSF9* was strongly negatively correlated with immunostimulators *C10* or *f54* and strongly negatively correlated with immunosuppressants *CD244*, *PDCD1*, and *TGFB1*. This deepened the understanding of the signal pathway related to *IGSF9* in NPC and confirmed that *IGSF9* can be used as a suitable diagnostic and prognostic gene for the prognosis of NPC.

Identifying central genes and key pathways can highlight the development of NPC at the molecular level and can be used for diagnosis, prediction, and treatment research. The potential impact of these three marker genes on immunotherapy was explored, namely, the correlation analysis with immunomodulatory factors. The results demonstrated that it was significantly correlated with 8 immunosuppressive factors and 11 immune activators. In other words, these three diagnostic markers are closely related to the immune response and immunotherapy of patients with NPC. Thus, the exact role of those 3 biomarkers in the immunomodulatory signaling pathways should be further explored in the next step, so as to help develop a novel strategy for the application of those biomarkers in the immunotherapy of NPC.

There are several limitations to this study. First, the data source of this study is single, and the amount of data included is not large, leading to some deviation in the analysis results. Second, our study is a retrospective study, and more prospective studies are required to verify the prognostic function of immune microenvironment-related signals. Third, although these three NPC-related genes are involved in many immune-related biological processes and signaling pathways, further functional tests should be performed to complement and clarify their specific roles in cellular signaling regulation.

In conclusion, the potential molecular mechanism of immune cell infiltration-related genes regulating NPC was explored; *FCER2*, *KHDRBS2*, and *IGSF9* were identified as diagnostic markers related to immune cell infiltration in NPC for the first time; their relationship with immune regulatory factors was analyzed. Our findings would facilitate diagnosis and guide the treatment of NPC.

## Figures and Tables

**Table 1:** Basic clinical information of 10 patients with NPC.

Order	Sex	Age (year)	T classification	N classification	M classification	Clinical stage
1	Male	39	T1	N1	M0	II
2	Female	69	T3	N3	M0	IVa
3	Male	42	T2	N2	M0	III
4	Male	57	T1	N2	M0	III
5	Male	47	T2	N1	M0	II
6	Female	15	N2	N2	M0	III
7	Male	43	T1	N2	M0	III
8	Male	49	T1	N2	M0	III
9	Male	72	T1	N1	M0	II
10	Male	54	T1	N0	M0	I

**Table 2:** RT-qPCR primers used in the current study.

Genes	Forward	Reverse
FCER2	ATCTGGGTGGATGGGAG	CAAGAGTGGAGAGGGGC
KHDRBS2	TAAATGGCTCAGAGGACT	CCGTATCAAAAAACAAGG
IGSF9	CCCTGAAGACGATTTTG	TGGGGGTCCTAGCACTA
GAPDH	CCCATCACCATCTTCCAGG	CATCACGCCACAGTTTCCC

## Data Availability

The data that support the findings of this study are available from the corresponding author upon reasonable request.

## Supplementary Materials

Supplementary Materials GO and KEGG pathway analyses were conducted to investigate the biological function of hub genes. Table S1: biological processes (BP), cellular components (CC), and molecular functions (MF). Table S2: KEGG pathways.
