# Supplementary material for: Differentially Infiltrated Identification of Novel Diagnostic Biomarkers Associated with Immune Infiltration in Nasopharyngeal Carcinoma
Source: Dis Markers. 2022 Nov 17;2022:3934704. doi: 10.1155/2022/3934704 (PMC9691307; doi:10.1155/2022/3934704)
Supplement: Supplementary Materials — GO and KEGG pathway analyses were conducted to investigate the biological function of hub genes. Table S1: biological processes (BP), cellular components (CC), and molecular functions (MF). Table S2: KEGG pathways. [file 3934704.f1.zip › 3934704.f1/Table S1 (1).pdf]

ON	ID	Descript	GeneRati	BgRatio	pvalue	p.adjust	qvalue	geneID	Cou
BP	GO:0042113	B cell activation	31/248	328/18866	6.73E-18	1.98E-14	1.78E-14	IGHD/BLK/FCRL1/MS4A1/CD22/CD79B/BANK1/CD19/GAPT/HHEX/LYL1/CR2/MEF2C/FCRL3/PRKCB/TNFRSF13C/TNFRSF13B/POU2F2/TBC1D10C/BTK/CXCR5/PTPN6/CD40LG/MFNG/IKZF3/PIK3CD/CD28/DOCK11/CMTM7/ZFP36L1/INPP5D	31
BP	GO:0030098	lymphocyte differentiation	30/248	368/18866	1.38E-15	2.02E-12	1.82E-12	MS4A1/CD79B/CD19/GPR18/CR1/HHEX/TCF7/LYL1/CR2/FCRL3/LY9/DTX1/POU2F2/BTK/PTPN6/CD40LG/TGFBR2/IKZF1/RHOH/MFNG/IKZF3/PIK3CD/FOXP1/CD28/LCK/DOCK11/CMTM7/ZFP36L1/INPP5D/MDK	30
BP	GO:0002429	immune response-activating cell	31/248	481/18866	2.70E-13	1.99E-10	1.79E-10	IGHD/BLK/MS4A1/CD22/CD79B/CD19/CR1/CR2/MEF2C/FCRL3/PRKCB/TXK/NCR3/BTK/PTPN6/PVRIG/CSK/CYFIP2/PIK3CD/FOXP1/CD28/LCK/SKAP1/LAT/PAX5/ELMO1/WAS/INPP5D/FYN/CYFIP1/MYO10	31
BP	GO:0002757	immune response-activating signal	31/248	481/18866	2.70E-13	1.99E-10	1.79E-10	IGHD/BLK/MS4A1/CD22/CD79B/CD19/CR1/CR2/MEF2C/FCRL3/PRKCB/TXK/NCR3/BTK/PTPN6/PVRIG/CSK/CYFIP2/PIK3CD/FOXP1/CD28/LCK/SKAP1/LAT/PAX5/ELMO1/WAS/INPP5D/FYN/CYFIP1/MYO10	31
BP	GO:0030183	B cell differentiation	17/248	137/18866	3.13E-12	1.84E-09	1.65E-09	MS4A1/CD79B/CD19/HHEX/LYL1/CR2/FCRL3/POU2F2/BTK/PTPN6/CD40LG/MFNG/IKZF3/DOCK11/CMTM7/ZFP36L1/INPP5D	17
BP	GO:0050851	antigen receptor-mediate	24/248	325/18866	9.22E-12	4.51E-09	4.06E-09	IGHD/BLK/MS4A1/CD22/CD79B/CD19/MEF2C/FCRL3/PRKCB/TXK/BTK/PTPN6/PVRIG/CSK/PIK3CD/FOXP1/CD28/LCK/SKAP1/LAT/PAX5/WAS/INPP5D/FYN	24
BP	GO:0046651	lymphocyte proliferation	22/248	283/18866	2.59E-11	9.94E-09	8.93E-09	BLK/MS4A1/CD22/CD19/GAPT/CR2/MEF2C/FCRL3/TNFRSF13C/CLECL1/TNFRSF13B/CORO1A/BTK/PTPN6/CD40LG/TGFBR2/IKZF3/CD28/RASAL3/RAC2/INPP5D/FYN	22
BP	GO:0070661	leukocyte proliferation	23/248	313/18866	2.82E-11	9.94E-09	8.93E-09	BLK/MS4A1/CD22/CD19/GAPT/HHEX/CR2/MEF2C/FCRL3/TNFRSF13C/CLECL1/TNFRSF13B/CORO1A/BTK/PTPN6/CD40LG/TGFBR2/IKZF3/CD28/RASAL3/RAC2/INPP5D/FYN	23
BP	GO:0032943	mononuclear cell prolifera	22/248	286/18866	3.19E-11	9.94E-09	8.93E-09	BLK/MS4A1/CD22/CD19/GAPT/CR2/MEF2C/FCRL3/TNFRSF13C/CLECL1/TNFRSF13B/CORO1A/BTK/PTPN6/CD40LG/TGFBR2/IKZF3/CD28/RASAL3/RAC2/INPP5D/FYN	22
BP	GO:0042100	B cell proliferation	14/248	97/18866	3.38E-11	9.94E-09	8.93E-09	BLK/MS4A1/CD22/CD19/GAPT/CR2/MEF2C/FCRL3/TNFRSF13C/TNFRSF13B/BTK/CD40LG/IKZF3/INPP5D	14
BP	GO:0042110	T cell activation	27/248	483/18866	2.60E-10	6.93E-08	6.23E-08	GPR18/CR1/TCF7/BTLA/LY9/TNFRSF13C/DTX1/CLECL1/CORO1A/PTPN6/CD40LG/TGFBR2/CSK/RHOH/PIK3CD/APBB1IP/FOXP1/CD28/RASAL3/LCK/ITGAL/RAC2/LAT/ZFP36L1/WAS/FYN/MDK	27
BP	GO:0050853	B cell receptor signalin	15/248	135/18866	3.00E-10	7.34E-08	6.60E-08	IGHD/BLK/MS4A1/CD22/CD79B/CD19/MEF2C/FCRL3/PRKCB/BTK/PTPN6/PIK3CD/FOXP1/LCK/PAX5	15
BP	GO:0070663	regulation of leukocyt	18/248	240/18866	3.15E-09	7.11E-07	6.39E-07	BLK/CD22/HHEX/MEF2C/FCRL3/TNFRSF13C/CLECL1/TNFRSF13B/CORO1A/BTK/PTPN6/CD40LG/TGFBR2/IKZF3/CD28/RASAL3/RAC2/INPP5D	18
BP	GO:0050670	regulation of lympho	17/248	219/18866	5.20E-09	1.09E-06	9.81E-07	BLK/CD22/MEF2C/FCRL3/TNFRSF13C/CLECL1/TNFRSF13B/CORO1A/BTK/PTPN6/CD40LG/TGFBR2/IKZF3/CD28/RASAL3/RAC2/INPP5D	17

BP	GO:0032944	regulation of mononu	17/248	221/18866	5.96E-09	1.16E-06	1.04E-06	BLK/CD22/MEF2C/FCRL3/TNFRSF13C/CLECL1/TNFRSF13B/CORO1A/BTK/PTPN6/CD40LG/TGFBR2/IKZF3/CD28/RASAL3/RAC2/INPP5D	17
BP	GO:0007265	Ras protein signal transdu	21/248	346/18866	6.69E-09	1.16E-06	1.04E-06	RASGRP2/GPR18/RALGPS2/RASGRP3/ARHGAP4/RHOH/ARHGEF1/PSD4/RASAL3/RAC2/ARHGAP17/LAT/RASA3/ELMO1/WAS/ARHGEF18/RASSF1/CYTH1/RASA2/CYFIP1/RTKN	21
BP	GO:0050864	regulation of B cell	16/248	195/18866	6.72E-09	1.16E-06	1.04E-06	IGHD/BLK/CD22/BANK1/CD19/MEF2C/FCRL3/TNFRSF13C/TNFRSF13B/TBC1D10C/BTK/PTPN6/IKZF3/CD28/ZFP36L1/INPP5D	16
BP	GO:0051056	regulation of small GTPase	20/248	323/18866	1.11E-08	1.81E-06	1.63E-06	GPR18/RALGPS2/ARHGAP9/SIPA1/ARHGAP4/RHOH/ARHGAP15/ARHGEF1/PSD4/RASAL3/FGD3/RAC2/ARHGAP17/RASA3/GMIP/ARHGEF18/MYO9A/CYTH1/SOS2/RASA2	20
BP	GO:0007159	leukocyte cell-cell	21/248	364/18866	1.62E-08	2.51E-06	2.26E-06	SELL/BTLA/TNFRSF13C/DTX1/CLECL1/CORO1A/PTPN6/CD40LG/TGFBR2/CSK/RHOH/CD28/RASAL3/LCK/SKAP1/ITGAL/RAC2/ETS1/STK10/FYN/MDK	21
BP	GO:0050854	regulation of	10/248	69/18866	2.21E-08	3.25E-06	2.92E-06	BLK/CD22/CD19/FCRL3/PRKCB/PTPN6/PVRIG/FOXP1/LCK/PAX5	10
BP	GO:0050855	regulation of B	7/248	29/18866	7.60E-08	1.06E-05	9.56E-06	BLK/CD22/CD19/FCRL3/PRKCB/FOXP1/PAX5	7
BP	GO:0022407	regulation of cell-cell adhesio	22/248	439/18866	8.95E-08	1.14E-05	1.03E-05	BLK/BTLA/TNFRSF13C/DTX1/CLECL1/CORO1A/PTPN6/CD40LG/TGFBR2/CSK/RHOH/CD28/RASAL3/LCK/SKAP1/FXYD5/ETS1/AKNA/FYN/CELSR2/MDK/MYO10	22
BP	GO:0050863	regulation of T cell	19/248	332/18866	9.11E-08	1.14E-05	1.03E-05	CR1/TCF7/BTLA/TNFRSF13C/DTX1/CLECL1/CORO1A/PTPN6/CD40LG/TGFBR2/CSK/RHOH/CD28/RASAL3/LCK/RAC2/LAT/FYN/MDK	19
BP	GO:1903039	positive regulation of	16/248	235/18866	9.32E-08	1.14E-05	1.03E-05	BTLA/TNFRSF13C/CLECL1/CORO1A/PTPN6/CD40LG/TGFBR2/CSK/RHOH/CD28/RASAL3/LCK/SKAP1/ETS1/FYN/MDK	16
BP	GO:0030888	regulation of B	9/248	62/18866	1.12E-07	1.32E-05	1.18E-05	BLK/CD22/MEF2C/FCRL3/TNFRSF13C/TNFRSF13B/BTK/IKZF3/INPP5D	9
BP	GO:0022409	positive regulation of	17/248	279/18866	1.82E-07	2.06E-05	1.85E-05	BTLA/TNFRSF13C/CLECL1/CORO1A/PTPN6/CD40LG/TGFBR2/CSK/RHOH/CD28/RASAL3/LCK/SKAP1/ETS1/FYN/MDK/MYO10	17
BP	GO:0051251	positive regulation of	19/248	357/18866	2.81E-07	3.05E-05	2.75E-05	IGHD/MEF2C/FCRL3/BTLA/TNFRSF13C/CLECL1/CORO1A/BTK/PTPN6/CD40LG/TGFBR2/CSK/RHOH/CD28/RASAL3/LCK/INPP5D/FYN/MDK	19
BP	GO:0031295	T cell costimul	8/248	56/18866	6.56E-07	6.89E-05	6.19E-05	BTLA/TNFRSF13C/PTPN6/CD40LG/CSK/CD28/LCK/FYN	8
BP	GO:0045619	regulation of	13/248	181/18866	8.37E-07	8.48E-05	7.62E-05	CR1/TCF7/FCRL3/DTX1/BTK/PTPN6/TGFBR2/RHOH/IKZF3/CD28/ZFP36L1/INPP5D/MDK	13
BP	GO:0031294	lymphocyte	8/248	58/18866	8.66E-07	8.48E-05	7.62E-05	BTLA/TNFRSF13C/PTPN6/CD40LG/CSK/CD28/LCK/FYN	8
BP	GO:0050870	positive regulation of T	14/248	214/18866	9.73E-07	9.22E-05	8.29E-05	BTLA/TNFRSF13C/CLECL1/CORO1A/PTPN6/CD40LG/TGFBR2/CSK/RHOH/CD28/RASAL3/LCK/FYN/MDK	14
BP	GO:0043087	regulation of GTPase activity	21/248	481/18866	1.67E-06	0.000153	0.000138	RASGRP2/TBC1D10C/S1PR1/RASGRP3/ARHGAP9/SIPA1/ARHGAP4/RHOH/ARHGAP15/ARHGEF1/RASAL3/FGD3/DOCK11/RABEP2/ARHGAP17/RASA3/GMIP/RIN3/MYO9A/RASA2/RTKN	21
BP	GO:0045577	regulation of B	6/248	29/18866	1.79E-06	0.000156	0.000141	FCRL3/BTK/PTPN6/IKZF3/ZFP36L1/INPP5D	6

BP	GO:1903037	regulation of leukocyt	17/248	329/18866	1.81E-06	0.000156	0.000141	BTLA/TNFRSF13C/DTX1/CLECL1/CORO1A/PTPN6/CD40LG/TGFBR2/CSK/RHOH/CD28/RASAL3/LCK/SKAP1/ETS1/FYN/MDK	17
BP	GO:0002696	positive regulation of	19/248	406/18866	1.94E-06	0.000163	0.000146	IGHD/MEF2C/FCRL3/BTLA/TNFRSF13C/CLECL1/CORO1A/BTK/PTPN6/CD40LG/TGFBR2/CSK/RHOH/CD28/RASAL3/LCK/INPP5D/FYN/MDK	19
BP	GO:0050867	positive regulation of	19/248	421/18866	3.29E-06	0.000262	0.000236	IGHD/MEF2C/FCRL3/BTLA/TNFRSF13C/CLECL1/CORO1A/BTK/PTPN6/CD40LG/TGFBR2/CSK/RHOH/CD28/RASAL3/LCK/INPP5D/FYN/MDK	19
BP	GO:0050869	negative regulati	6/248	32/18866	3.30E-06	0.000262	0.000236	BLK/BANK1/TNFRSF13B/TBC1D10C/BTK/INPP5D	6
BP	GO:0045785	positive regulation of	19/248	428/18866	4.18E-06	0.000323	0.00029	BTLA/TNFRSF13C/CLECL1/CORO1A/PTPN6/CD40LG/TGFBR2/CSK/RHOH/APBB1IP/CD28/RASAL3/LCK/SKAP1/ETS1/STK4/FYN/MDK/MYO10	19
BP	GO:0002115	store-operate	5/248	21/18866	6.46E-06	0.000487	0.000437	MS4A1/STIM1/HOMER1/HOMER3/STC2	5
BP	GO:0002285	lymphocyte	12/248	187/18866	7.14E-06	0.000524	0.000471	CD19/GAPT/LY9/CORO1A/CD40LG/MFNG/APBB1IP/FOXP1/CD28/ITGAL/DOCK11/MDK	12
BP	GO:1902105	regulation of leukocyt	15/248	290/18866	7.32E-06	0.000525	0.000472	CR1/TCF7/FCRL3/DTX1/BTK/PTPN6/TGFBR2/RHOH/IKZF3/FOXP1/CD28/ZFP36L1/INPP5D/TCTA/MDK	15
BP	GO:0043547	positive regulation of GTPase	18/248	406/18866	7.66E-06	0.000536	0.000482	RASGRP2/TBC1D10C/S1PR1/RASGRP3/ARHGAP9/SIPA1/ARHGAP4/ARHGAP15/ARHGEF1/RASAL3/DOCK11/RABEP2/ARHGAP17/RASA3/GMIP/RIN3/MYO9A/RASA2	18
BP	GO:0002460	adaptive immune respons	17/248	370/18866	8.66E-06	0.000592	0.000532	IGHD/FCER2/CD19/BACH2/GAPT/CR1/CR2/MEF2C/LY9/TNFRSF13C/POU2F2/BTK/PTPN6/CD40LG/CD28/WAS/INPP5D	17
BP	GO:0045576	mast cell	7/248	61/18866	1.49E-05	0.000992	0.000892	BLK/CNR2/BTK/RHOH/PIK3CD/RAC2/LAT	7
BP	GO:0002260	lymphocyte	7/248	62/18866	1.66E-05	0.001081	0.000972	GAPT/MEF2C/TNFRSF13C/TNFRSF13B/CORO1A/DOCK11/LAT	7
BP	GO:0001776	leukocyte	8/248	86/18866	1.75E-05	0.001116	0.001003	GAPT/MEF2C/TNFRSF13C/TNFRSF13B/CORO1A/PIK3CD/DOCK11/LAT	8
BP	GO:0050852	T cell receptor	12/248	206/18866	1.89E-05	0.001183	0.001064	TXK/PTPN6/PVRIG/CSK/PIK3CD/CD28/LCK/SKAP1/LAT/WAS/INPP5D/FYN	12
BP	GO:0002335	mature B cell	5/248	28/18866	2.89E-05	0.001771	0.001592	CD19/POU2F2/MFNG/DOCK11/CMTM7	5
BP	GO:0030217	T cell different	13/248	253/18866	3.21E-05	0.001924	0.001729	GPR18/CR1/TCF7/LY9/DTX1/TGFBR2/RHOH/PIK3CD/FOXP1/CD28/LCK/ZFP36L1/MDK	13
BP	GO:0002695	negative regulati	11/248	184/18866	3.33E-05	0.001954	0.001757	BLK/CNR2/BANK1/DTX1/TNFRSF13B/TBC1D10C/BTK/PTPN6/TSPAN32/INPP5D/MDK	11
BP	GO:0001782	B cell homeos	5/248	29/18866	3.46E-05	0.001993	0.001791	GAPT/MEF2C/TNFRSF13C/TNFRSF13B/DOCK11	5
BP	GO:2001256	regulation of	4/248	15/18866	3.55E-05	0.002006	0.001803	STIM1/HOMER1/HOMER3/STC2	4
BP	GO:0048872	homeostasis of number	13/248	256/18866	3.63E-05	0.00201	0.001807	GAPT/MEF2C/TNFRSF13C/TNFRSF13B/CORO1A/IKZF1/PIK3CD/KLF2/DOCK11/ETS1/LAT/ZFP36L1/INPP5D	13
BP	GO:0002683	negative regulation of	18/248	463/18866	4.40E-05	0.002392	0.00215	BLK/CD22/CNR2/BANK1/GPR18/CR1/FCRL3/DTX1/TNFRSF13B/TBC1D10C/BTK/PTPN6/PVRIG/TSPAN32/ZFP36L1/INPP5D/TCTA/MDK	18
BP	GO:0016064	immunoglobulin	12/248	227/18866	4.91E-05	0.002623	0.002358	IGHD/FCER2/CD19/GAPT/CR1/CR2/POU2F2/BTK/PTPN6/CD40LG/CD28/INPP5D	12
BP	GO:0019724	B cell mediate	12/248	230/18866	5.58E-05	0.002925	0.002629	IGHD/FCER2/CD19/GAPT/CR1/CR2/POU2F2/BTK/PTPN6/CD40LG/CD28/INPP5D	12

BP	GO:0030889	negative regulati	4/248	17/18866	6.06E-05	0.003125	0.002809	BLK/TNFRSF13B/BTK/INPP5D	4
BP	GO:0046777	protein autopho	12/248	237/18866	7.43E-05	0.003766	0.003385	BLK/TXK/BTK/MAP4K1/CSK/LCK/STK17B/STK10/PPP2R5C/STK4/FYN/TNKS1BP1	12
BP	GO:0002819	regulation of	10/248	168/18866	7.86E-05	0.003899	0.003505	FCER2/CR1/MEF2C/TNFRSF13C/BTK/PTPN6/CD48/CD28/SKAP1/WAS	10
BP	GO:0050671	positive regulati	9/248	136/18866	7.96E-05	0.003899	0.003505	MEF2C/FCRL3/TNFRSF13C/CLECL1/CORO1A/CD40LG/TGFBR2/CD28/RASAL3	9
BP	GO:0032946	positive regulati	9/248	137/18866	8.43E-05	0.004058	0.003648	MEF2C/FCRL3/TNFRSF13C/CLECL1/CORO1A/CD40LG/TGFBR2/CD28/RASAL3	9
BP	GO:0050866	negative regulati	11/248	207/18866	9.66E-05	0.004576	0.004114	BLK/CNR2/BANK1/DTX1/TNFRSF13B/TBC1D10C/BTK/PTPN6/TSPAN32/INPP5D/MDK	11
BP	GO:0007204	positive regulati	14/248	322/18866	9.81E-05	0.004576	0.004114	MS4A1/CD19/GPR18/P2RX5/CD52/CORO1A/CXCR5/PTPN6/S1PR1/S1PR4/LCK/RASA3/FYN/LRP6	14
BP	GO:0002449	lymphocyte mediate	15/248	366/18866	0.0001073	0.004927	0.004429	IGHD/FCER2/CD19/GAPT/CR1/CR2/CORO1A/POU2F2/NCR3/BTK/PTPN6/CD40LG/CD28/WAS/INPP5D	15
BP	GO:1903706	regulation of hemopo	18/248	498/18866	0.0001111	0.005023	0.004515	CR1/TCF7/MEF2C/FCRL3/PRKCB/DTX1/BTK/PTPN6/TGFBR2/RHOH/IKZF3/FOXP1/CD28/ETS1/ZFP36L1/INPP5D/TCTA/MDK	18
BP	GO:0006874	cellular calcium ion	17/248	456/18866	0.0001192	0.005308	0.004771	MS4A1/CD19/GPR18/PRKCB/P2RX5/CD52/CORO1A/CXCR5/PTPN6/S1PR1/S1PR4/LCK/RASA3/STIM1/FYN/LRP6/STC2	17
BP	GO:0016601	Rac protein	5/248	39/18866	0.0001507	0.006607	0.005939	RHOH/RAC2/ARHGAP17/ELMO1/CYFIP1	5
BP	GO:0055074	calcium ion homeos	17/248	468/18866	0.0001625	0.007022	0.006312	MS4A1/CD19/GPR18/PRKCB/P2RX5/CD52/CORO1A/CXCR5/PTPN6/S1PR1/S1PR4/LCK/RASA3/STIM1/FYN/LRP6/STC2	17
BP	GO:0070665	positive regulati	9/248	150/18866	0.0001684	0.007168	0.006444	MEF2C/FCRL3/TNFRSF13C/CLECL1/CORO1A/CD40LG/TGFBR2/CD28/RASAL3	9
BP	GO:0006816	calcium ion transpor	16/248	426/18866	0.0001737	0.007289	0.006552	MS4A1/CD19/FCRL3/PRKCB/P2RX5/CORO1A/PTPN6/CACNA1I/LCK/IL16/RASA3/STIM1/FYN/HOMER1/HOMER3/STC2	16
BP	GO:0051250	negative regulati	9/248	154/18866	0.0002052	0.008492	0.007634	BLK/BANK1/DTX1/TNFRSF13B/TBC1D10C/BTK/PTPN6/INPP5D/MDK	9
BP	GO:0008360	regulation of	9/248	156/18866	0.000226	0.009223	0.008291	CORO1A/RHOH/ARHGAP15/FGD3/RAC2/ARHGEF18/FYN/CYFIP1/MYO10	9
BP	GO:0042098	T cell prolifera	10/248	195/18866	0.0002664	0.010722	0.009638	TNFRSF13C/CLECL1/CORO1A/PTPN6/CD40LG/TGFBR2/CD28/RASAL3/RAC2/FYN	10
BP	GO:0051480	regulation of	14/248	357/18866	0.0002854	0.011332	0.010186	MS4A1/CD19/GPR18/P2RX5/CD52/CORO1A/CXCR5/PTPN6/S1PR1/S1PR4/LCK/RASA3/FYN/LRP6	14
BP	GO:0072503	cellular divalent inorgani	17/248	492/18866	0.0002918	0.011431	0.010276	MS4A1/CD19/GPR18/PRKCB/P2RX5/CD52/CORO1A/CXCR5/PTPN6/S1PR1/S1PR4/LCK/RASA3/STIM1/FYN/LRP6/STC2	17
BP	GO:0048010	vascular endothe	7/248	98/18866	0.0003121	0.012064	0.010845	HHEX/PRKCB/CYFIP2/ELMO1/FYN/CYFIP1/GRB10	7
BP	GO:0043303	mast cell	5/248	47/18866	0.0003686	0.013957	0.012546	BLK/BTK/PIK3CD/RAC2/LAT	5
BP	GO:0042129	regulation of T	9/248	167/18866	0.0003743	0.013957	0.012546	TNFRSF13C/CLECL1/CORO1A/PTPN6/CD40LG/TGFBR2/CD28/RASAL3/RAC2	9
BP	GO:0042102	positive regulati	7/248	101/18866	0.0003753	0.013957	0.012546	TNFRSF13C/CLECL1/CORO1A/CD40LG/TGFBR2/CD28/RASAL3	7
BP	GO:0002279	mast cell	5/248	48/18866	0.000407	0.014949	0.013438	BLK/BTK/PIK3CD/RAC2/LAT	5
BP	GO:0002448	mast cell	5/248	49/18866	0.0004485	0.016203	0.014566	BLK/BTK/PIK3CD/RAC2/LAT	5

BP	GO:0002923	regulation of	3/248	12/18866	0.0004522	0.016203	0.014566	FCER2/CR1/PTPN6	3
BP	GO:0032418	lysosome	6/248	75/18866	0.0004585	0.016229	0.014589	BLK/BTK/PIK3CD/TFEB/RAC2/LAT	6
BP	GO:0002703	regulation of	10/248	211/18866	0.0004974	0.017397	0.015639	BLK/FCER2/DNASE1L3/CR1/NCR3/BTK/PTPN6/CD28/RAC2/WAS	10
BP	GO:0002697	regulation of immune	16/248	470/18866	0.0005157	0.017824	0.016023	BLK/FCER2/CD22/DNASE1L3/CD19/CR1/CR2/FCRL3/NCR3/BTK/PTPN6/CD40LG/TSPAN32/CD28/RAC2/WAS	16
BP	GO:0070838	divalent metal ion	16/248	471/18866	0.0005277	0.018026	0.016204	MS4A1/CD19/FCRL3/PRKCB/P2RX5/CORO1A/PTPN6/CACNA1I/LCK/IL16/RASA3/STIM1/FYN/HOMER1/HOMER3/STC2	16
BP	GO:0050690	regulation of	4/248	29/18866	0.0005345	0.01805	0.016226	CD28/LCK/ELMO1/FYN	4
BP	GO:0051924	regulation of	11/248	253/18866	0.0005447	0.018187	0.016349	CD19/FCRL3/P2RX5/CORO1A/PTPN6/IL16/STIM1/FYN/HOMER1/HOMER3/STC2	11
BP	GO:0007492	endoderm	6/248	79/18866	0.0006059	0.019927	0.017913	ZFP36L1/SETD2/BMPR1A/LAMB1/COL5A1/COL11A1	6
BP	GO:0051125	regulation of	4/248	30/18866	0.0006104	0.019927	0.017913	CORO1A/GMFG/WAS/CYFIP1	4
BP	GO:0072511	divalent inorganic cation	16/248	478/18866	0.0006183	0.019963	0.017945	MS4A1/CD19/FCRL3/PRKCB/P2RX5/CORO1A/PTPN6/CACNA1I/LCK/IL16/RASA3/STIM1/FYN/HOMER1/HOMER3/STC2	16
BP	GO:0002312	B cell activatio	6/248	80/18866	0.0006479	0.020691	0.0186	CD19/GAPT/CD40LG/MFNG/CD28/DOCK11	6
BP	GO:0002431	Fc receptor	8/248	145/18866	0.0006753	0.021335	0.019178	BLK/CSK/CYFIP2/ELMO1/WAS/FYN/CYFIP1/MYO10	8
BP	GO:0050858	negative regulati	4/248	31/18866	0.0006937	0.021681	0.01949	CD22/FCRL3/PTPN6/PVRIG	4
BP	GO:0007599	hemostasis	13/248	348/18866	0.0007368	0.022786	0.020483	SERPINA5/BLK/PRKCB/P2RX5/TXK/PTPN6/CD40LG/FLI1/TSPAN32/LCK/DOCK11/WAS/FYN	13
BP	GO:0021591	ventricular	4/248	32/18866	0.0007847	0.024014	0.021586	PAX5/MECP2/CELSR2/TSKU	4
BP	GO:0002712	regulation of B	5/248	56/18866	0.0008339	0.024999	0.022472	FCER2/CR1/BTK/PTPN6/CD28	5
BP	GO:0002889	regulation of	5/248	56/18866	0.0008339	0.024999	0.022472	FCER2/CR1/BTK/PTPN6/CD28	5
BP	GO:2000106	regulation of	6/248	86/18866	0.0009496	0.028172	0.025325	BLK/MEF2C/BTK/PTCRA/PIK3CD/FOXP1	6
BP	GO:0002822	regulation of adaptive	8/248	153/18866	0.0009589	0.028172	0.025325	FCER2/CR1/MEF2C/TNFRSF13C/BTK/PTPN6/CD28/WAS	8
BP	GO:0032956	regulation of actin	13/248	360/18866	0.0010047	0.029225	0.026271	MEF2C/CORO1A/S1PR1/VILL/RHOH/CYFIP2/GMFG/RAC2/ARHGAP17/WAS/ARHGEF18/CYFIP1/MDK	13
BP	GO:0030595	leukocyte	10/248	232/18866	0.0010321	0.029727	0.026723	CNR2/GPR18/CORO1A/CXCR5/S1PR1/PIK3CD/IL16/RAC2/IL6R/MDK	10
BP	GO:0046578	regulation of	9/248	194/18866	0.0010966	0.031279	0.028118	GPR18/RALGPS2/PSD4/RASAL3/ARHGAP17/RASA3/ARHGEF18/CYTH1/RASA2	9
BP	GO:0098751	bone cell	4/248	35/18866	0.0011078	0.031296	0.028132	PTPN6/FLI1/FOXP1/PIP4K2A	4
BP	GO:0030168	platelet activatio	8/248	158/18866	0.0011798	0.032702	0.029397	BLK/PRKCB/TXK/PTPN6/CD40LG/TSPAN32/LCK/FYN	8
BP	GO:0060402	calcium ion	8/248	158/18866	0.0011798	0.032702	0.029397	MS4A1/CD19/P2RX5/CORO1A/PTPN6/LCK/RASA3/FYN	8

BP	GO:0002381	immunoglobulin	5/248	61/18866	0.0012315	0.033813	0.030396	GAPT/POU2F2/BTK/CD40LG/CD28	5
BP	GO:0046849	bone remodel	6/248	91/18866	0.0012751	0.034689	0.031183	S1PR1/CSK/RAC2/INPP5D/LRP6/MDK	6
BP	GO:0035855	megakaryocyte	3/248	17/18866	0.0013315	0.03589	0.032262	PTPN6/FLI1/PIP4K2A	3
BP	GO:0030224	monocyte	4/248	38/18866	0.0015144	0.040083	0.036032	MEF2C/FOXP1/ZFP36L1/INPP5D	4
BP	GO:1903131	mononuclear	4/248	38/18866	0.0015144	0.040083	0.036032	MEF2C/FOXP1/ZFP36L1/INPP5D	4
BP	GO:0006909	phagocytosis	13/248	382/18866	0.0017081	0.044806	0.040277	IGHD/PLD4/CORO1A/CSK/RHOH/CYFIP2/ITGAL/RAC2/ELMO1/WAS/FYN/CYFIP1/MYO10	13
BP	GO:0030099	myeloid cell different	14/248	431/18866	0.0017736	0.046114	0.041453	MEF2C/PRKCB/PTPN6/TGFBR2/IKZF1/PIK3CD/FLI1/FOXP1/KLF2/ETS1/PIP4K2A/ZFP36L1/INPP5D/TCTA	14
BP	GO:0007015	actin filament organiz	14/248	434/18866	0.0018908	0.048731	0.043805	CORO1A/S1PR1/VILL/RHOH/CYFIP2/GMFG/RAC2/ARHGAP17/ELMO1/WAS/ARHGEF18/CYFIP1/MYO19/SHROOM2	14
CC	GO:0001772	immunological	7/264	39/19559	8.02E-07	0.000251	0.000235	CD37/CORO1A/RHOH/CD28/LCK/SKAP1/LAT	7
CC	GO:0045121	membrane raft	17/264	329/19559	2.62E-06	0.000285	0.000268	MS4A1/CD19/BTK/CD48/S1PR1/TGFBR2/LIPE/CSK/SLC34A1/LCK/SKAP1/LAT/INPP5D/STIM1/FYN/BMPR1A/LRP6	17
CC	GO:0098857	membrane microdo	17/264	330/19559	2.73E-06	0.000285	0.000268	MS4A1/CD19/BTK/CD48/S1PR1/TGFBR2/LIPE/CSK/SLC34A1/LCK/SKAP1/LAT/INPP5D/STIM1/FYN/BMPR1A/LRP6	17
CC	GO:0098589	membrane region	17/264	343/19559	4.58E-06	0.000359	0.000336	MS4A1/CD19/BTK/CD48/S1PR1/TGFBR2/LIPE/CSK/SLC34A1/LCK/SKAP1/LAT/INPP5D/STIM1/FYN/BMPR1A/LRP6	17
CC	GO:0009897	external side of plasma	18/264	417/19559	1.59E-05	0.000998	0.000936	IGHD/SERPINA5/MS4A1/FCER2/CD22/CD79B/CD19/SELL/TNFRSF13C/CXCR5/CD40LG/S1PR1/TGFBR2/CD28/GFRA4/IL6R/BMPR1A/PRLR	18
CC	GO:0044853	plasma membra	7/264	113/19559	0.0008631	0.045024	0.042246	MS4A1/TGFBR2/LIPE/SKAP1/STIM1/BMPR1A/LRP6	7
MF	GO:0005085	guanyl-nucleotide	17/251	215/18352	7.06E-09	3.21E-06	3.01E-06	RASGRP2/DENND1C/RALGPS2/RASGRP3/ARHGEF1/PSD4/SH2D3C/RGL4/FGD3/DOCK11/DENND2D/ELMO1/ARHGEF18/RIN3/CYTH1/SOS2/HERC1	17
MF	GO:0030695	GTPase regulator activity	18/251	307/18352	2.50E-07	5.68E-05	5.34E-05	TBC1D10C/RASGRP3/ARHGAP9/SIPA1/ARHGAP4/RHOH/ARHGAP15/ARHGEF1/RASAL3/RABEP2/ARHGAP17/RASA3/WAS/GMIP/RIN3/MYO9A/RASA2/RTKN	18
MF	GO:0060589	nucleoside-triphosphatase	18/251	348/18352	1.54E-06	0.000234	0.000219	TBC1D10C/RASGRP3/ARHGAP9/SIPA1/ARHGAP4/RHOH/ARHGAP15/ARHGEF1/RASAL3/RABEP2/ARHGAP17/RASA3/WAS/GMIP/RIN3/MYO9A/RASA2/RTKN	18
MF	GO:0017048	Rho GTPase binding	12/251	162/18352	2.42E-06	0.000274	0.000258	ARHGAP4/RHOH/CYFIP2/ARHGEF1/FGD3/DOCK11/ARHGAP17/ELMO1/WAS/ARHGEF18/CYFIP1/RTKN	12
MF	GO:0005096	GTPase activator activity	15/251	275/18352	6.19E-06	0.000562	0.000528	TBC1D10C/RASGRP3/ARHGAP9/SIPA1/ARHGAP4/ARHGAP15/ARHGEF1/RASAL3/RABEP2/ARHGAP17/RASA3/GMIP/RIN3/MYO9A/RASA2	15
MF	GO:0004715	non-membra	6/251	46/18352	3.65E-05	0.002763	0.002595	BLK/TXK/BTK/CSK/LCK/FYN	6

MF	GO:0017016	Ras GTPase binding	17/251	415/18352	6.13E-05	0.003975	0.003732	TBC1D10C/DENND1C/RASGRP3/ARHGAP4/RHOH/CYFIP2/ARHGEF1/FGD3/DOCK11/DENND2D/ARHGAP17/ELMO1/WAS/ARHGEF18/RIN3/CYFIP1/RTKN	17
MF	GO:0031267	small GTPase binding	17/251	428/18352	8.93E-05	0.00507	0.004761	TBC1D10C/DENND1C/RASGRP3/ARHGAP4/RHOH/CYFIP2/ARHGEF1/FGD3/DOCK11/DENND2D/ARHGAP17/ELMO1/WAS/ARHGEF18/RIN3/CYFIP1/RTKN	17
MF	GO:0005088	Ras guanyl-	8/251	115/18352	0.0001838	0.009274	0.008708	DENND1C/RASGRP3/ARHGEF1/FGD3/DOCK11/DENND2D/ARHGEF18/RIN3	8
MF	GO:0035256	G protein-	3/251	11/18352	0.0003846	0.01746	0.016396	FYN/HOMER1/HOMER3	3
MF	GO:0017124	SH3 domain	8/251	131/18352	0.000446	0.018407	0.017285	KHDRBS2/DTX1/PTPN6/SKAP1/ARHGAP17/ELMO1/WAS/INPP5D	8
MF	GO:0048365	Rac GTPase	6/251	73/18352	0.0004881	0.018466	0.01734	ARHGAP4/CYFIP2/ARHGAP17/ELMO1/WAS/CYFIP1	6
MF	GO:0019903	protein phosph	8/251	149/18352	0.0010404	0.036334	0.034118	CD22/FCRL2/FCRL3/CSK/LCK/SKAP1/FOXO1/EIF4EBP1	8
MF	GO:0019902	phosphatase	9/251	194/18352	0.0014407	0.04672	0.043871	CD22/FCRL2/FCRL3/PVRIG/CSK/LCK/SKAP1/FOXO1/EIF4EBP1	9
