# Supplementary material for: Differentially Infiltrated Identification of Novel Diagnostic Biomarkers Associated with Immune Infiltration in Nasopharyngeal Carcinoma
Source: Dis Markers. 2022 Nov 17;2022:3934704. doi: 10.1155/2022/3934704 (PMC9691307; doi:10.1155/2022/3934704)
Supplement: Supplementary Materials — GO and KEGG pathway analyses were conducted to investigate the biological function of hub genes. Table S1: biological processes (BP), cellular components (CC), and molecular functions (MF). Table S2: KEGG pathways. [file 3934704.f1.zip › 3934704.f1/Table S2 (1).pdf]

Table S2 KEGG pathways

ID	Description	GeneRatio	BgRatio	pvalue	p.adjust	qvalue	geneID	Count
hsa04662	B cell receptor signaling pathway	13/137	82/8106	8.54E-10	1.87E-07	1.60E-07	933/974/971/930/1380/5579/695/5777/25780/5293/5880/3635/6655	13
hsa04650	Natural killer cell mediated cytotoxicity	11/137	131/8106	1.21E-05	0.001327	0.001135	5579/259197/5777/962/5293/3932/3683/5880/27040/2534/6655	11
hsa05340	Primary immunodeficiency	6/137	38/8106	3.70E-05	0.002698	0.002308	930/115650/23495/695/959/3932	6
hsa04068	FoxO signaling pathway	10/137	131/8106	6.94E-05	0.003802	0.003253	1901/8698/7048/5293/10365/2308/6789/6655/9456/9454	10
hsa04664	Fc epsilon RI signaling pathway	7/137	68/8106	0.000139	0.005192	0.004442	695/5293/5880/27040/3635/2534/6655	7
hsa04014	Ras signaling pathway	13/137	232/8106	0.000142	0.005192	0.004442	10235/5579/25780/5293/64926/5880/2113/27040/22821/11186/6789/6655/5922	13
hsa04640	Hematopoietic cell lineage	8/137	99/8106	0.000255	0.007976	0.006824	931/2208/933/930/951/1378/1380/3570	8
hsa04660	T cell receptor signaling pathway	8/137	104/8106	0.000357	0.009783	0.00837	5777/959/5293/940/3932/27040/2534/6655	8
hsa04015	Rap1 signaling pathway	11/137	210/8106	0.000826	0.019282	0.016497	10235/5579/25780/6494/5293/54518/8631/3683/5880/27040/5330	11
hsa04071	Sphingolipid signaling pathway	8/137	119/8106	0.00088	0.019282	0.016497	5579/1901/8698/5293/5880/5330/5527/2534	8
hsa04611	Platelet activation	8/137	124/8106	0.001152	0.020639	0.017658	10235/695/9138/5293/54518/5330/6786/2534	8
hsa04520	Adherens junction	6/137	71/8106	0.001215	0.020639	0.017658	6932/5777/7048/5880/7454/2534	6
hsa04666	Fc gamma R-mediated phagocytosis	7/137	97/8106	0.001225	0.020639	0.017658	5579/5293/5880/27040/7454/3635/4651	7

hsa04062	Chemokine signaling pathway	10/137	192/8106	0.001489	0.023286	0.019923	10235/5579/643/5293/5880/2870/9844/7454/5330/6655	10
hsa04064	NF-kappa B signaling pathway	7/137	104/8106	0.001838	0.026829	0.022954	5579/115650/695/959/7188/3932/27040	7
hsa04670	Leukocyte transendothelial migration	7/137	114/8106	0.003097	0.042386	0.036264	5579/7294/6494/399/5293/3683/5880	7
hsa05416	Viral myocarditis	5/137	60/8106	0.003323	0.042811	0.036628	959/940/3683/5880/2534	5
hsa04010	MAPK signaling pathway	12/137	294/8106	0.004017	0.048875	0.041816	10235/4208/5579/25780/7048/11184/5871/8911/5880/6789/6655/5922	12
